# Publications on Clinical Research in Otolaryngology–A Systematic Analysis of Leading Journals in 2010

**DOI:** 10.3389/fsurg.2019.00018

**Published:** 2019-04-09

**Authors:** Nina M. Kaper, Geerte G. J. Ramakers, Mark C. J. Aarts, Geert J. M. G. van der Heijden

**Affiliations:** ^1^Department of Otolaryngology–Head and Neck Surgery, University Medical Center Utrecht, Utrecht, Netherlands; ^2^Department of Otolaryngology, Jeroen Bosch Hospital, s'-Hertogenbosch, Netherlands; ^3^Department of Social Dentistry, Academic Center for Dentistry Amsterdam, University of Amsterdam, VU University, Amsterdam, Netherlands

**Keywords:** evidence-based medicine, evidence-based practice, impact factor, otolaryngology, therapy, diagnosis, prognosis, etiology

## Abstract

**Background:** We wanted to asses and characterize the volume of Otolaryngology publications on clinical research, published in major journals.

**Methods and Material:** To assess volume and study type of clinical research in Otolaryngology we performed a literature search in high impact factor journals. We included 10 high impact factor Otolaryngology journals and 20 high impact factor medical journals outside this field (2011). We extracted original publications and systematic reviews from 2010. Publications were classified according to their research question, that is therapy, diagnosis, prognosis or etiology.

**Results:** From Otolaryngology journals (impact factor 1.8 to 2.8) we identified 694 (46%) publications on original observations and 27 (2%) systematic reviews. From selected medical journals (impact factor 6.0 to 101.8) 122 (2%) publications related to Otolaryngology, 102 (83%) were on original observations and 2 (0.04%) systematic reviews. The most common category was therapy (40%).

**Conclusion:** Half of publications in Otolaryngology concerns clinical research, which is higher than other specialties. In medical journals outside the field of Otolaryngology, a small proportion (2%) of publications is related to Otolaryngology. Striking is that systematic reviews, which are considered high level evidence, make up for only 2% of publications. We must ensure an increase of clinical research for optimizing medical practice.

## Introduction

Clinicians strive to provide evidence-based patient care (1). According to the principles of evidence-based medicine (EBM), they should evaluate all available research for the best evidence and combine this with their experience and patients' preferences (1, 2). Therefore, clinicians are in need of publications reporting on health outcomes in patients, that provide answers to clinical research questions. These studies are addressed to as clinical research and have therefore a direct possibility to influence clinical practice (1, 2). Other research types, such as biological experiments or individual clinician's experiences can also be important, but their impact on clinical practice is often limited (2).

We initiated this study because we wanted to asses and characterize the volume of Otolaryngology publications on clinical research. Four important categories in clinical research can be distinguished (see Table 1), on which we will emphasize in this study (3).

**Table 1:** Publication topic.

	**Description**
Therapy	Causally explains and predicts the course of disease as given by an intervention for therapy (including adverse effects studies), prevention, rehabilitation, quality improvement, or continuing medical education, and clinical or non-clinical profile
Diagnosis	Content pertains directly to using a tool to arrive at a diagnosis of a disease or condition.
Prognosis	Content pertains directly to the prediction of the clinical course or the natural history of a disease or condition with the disease or condition existing at the beginning of the study.
Etiology	Determines if there is a causal relation between an exposure and a disease or condition.
Other	Costs and economics, Qualitative, Test development and validation, Descriptive study, Product development

Besides original publications, systematic reviews are also important for decision making in patient care. They collect and summarize all existing publications and are considered the highest quality, i.e., level 1a, evidence (4).

In the past, similar studies have been performed, showing a constant amount of 77% clinical research in'69,'79, and'89 in four major Otolaryngology journals (5). In 1999, clinical research accounted for 72% of publications in four major journals (6). Six major Otolaryngology journals were reviewed for the years 1993 and 2003, showing an increase in clinical research from 72 to 73% (7).

The purpose of our study is to provide insight in the volume and type of clinical research that is published in 1 year, in the field of Otorhinolaryngology. In addition, we compare leading Otolaryngology specialty journals to journals outside this field, based on their impact factor (8).

## Materials and Methods

### Selection of Journals

**Figure 1:**
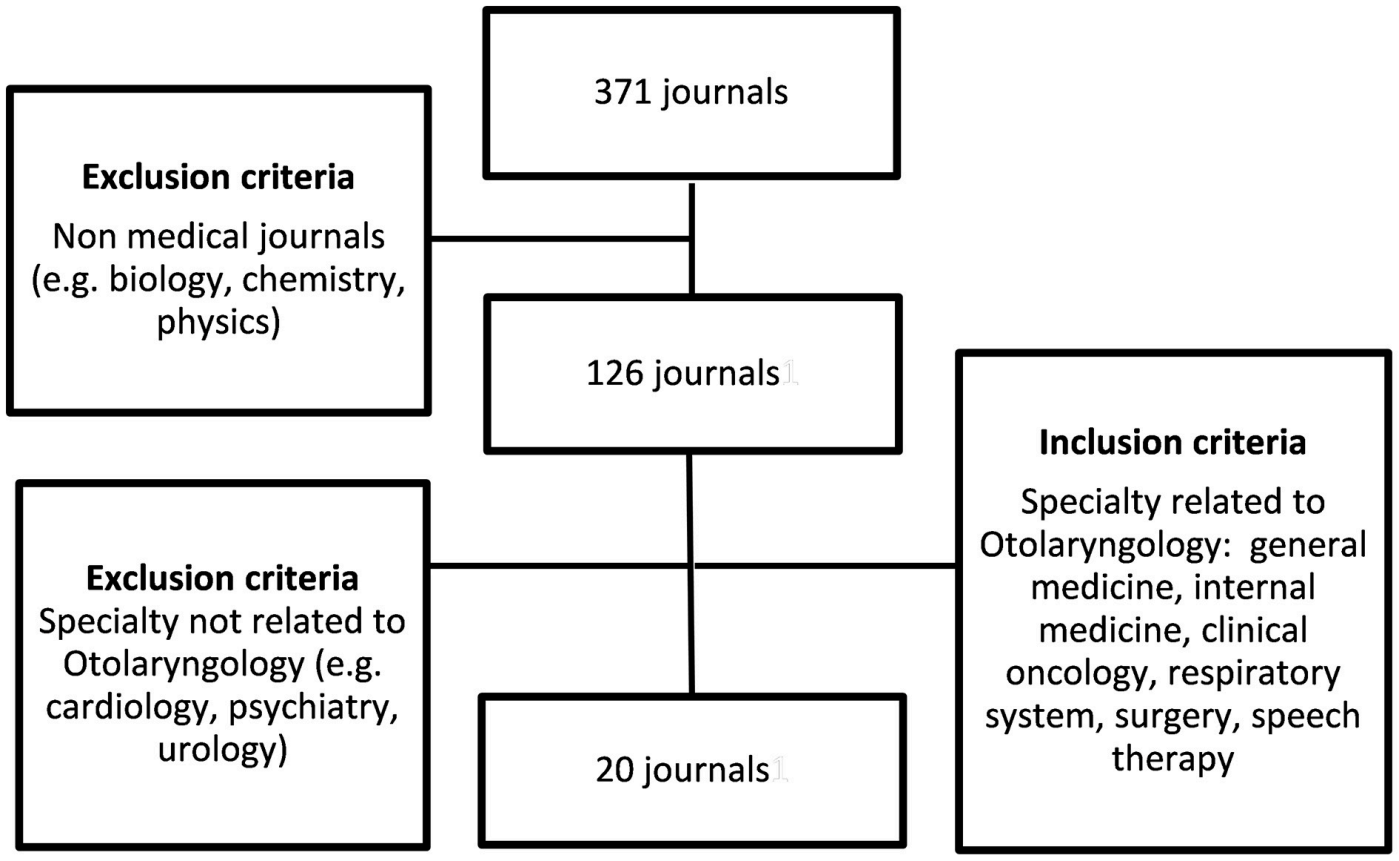
Selection of journals.

Data collection was carried out in February 2012. We identified leading journals by their impact factor. We selected the top 10 impact factor Otolaryngology journals, using the 2011 impact factor (8). We then searched for medical journals outside the field of Otolaryngology. We used the 2011 impact factor to rank journals from high to low and selected the first 20 journals that were likely to publish articles related to Otolaryngology. The journals were selected based on their scope. The in- and exclusion criteria can be found in Figure 1. The overview of all reviewed journals can be found online (Appendix 1). Two authors independently selected journals; initial disagreement was resolved by consensus (NK and GR).

### Selection of Publications

We retrieved full texts of all citable articles, i.e., peer-reviewed articles from the selected Otolaryngology journals. From the selected medical journals two authors independently retrieved full texts of all citable articles concerning Otolaryngology based on title or abstract, initial disagreement was resolved by consensus (NK and GR).

We selected all original publications on health outcomes in patients, with a determinant or outcome considered relevant for patient care (3). (For selection criteria see Table 2) We also selected systematic reviews and meta-analyses that were based on original publications, relevant for patient care. Case reports were excluded (4).

**Table 2:** Publication types.

	**Definition**
Citable article	All peer-reviewed publications (letters, editorials and symposium reports were excluded)
Original publication	Authors report first-hand observations
Clinical study	Study conducted in living healthy or affected patients, or in tissue/ body fluids of living humans, with a determinant and outcome relevant for patient care, reporting outcomes for 10 or more patients
Systematic review (and meta-analysis)	Authors systematically select, assess and synthesize all relevant original publications on a particular topic
Case report	Original publication that identifies personalized data (< 10 patients)

The selected studies were further classified based on the purpose of their research question, e.g., therapy, diagnosis, prognosis or etiology (see Table 1 for definitions) (3).

Two authors (NK and GR) independently retrieved and reviewed all publications. Initial disagreement on selection and categorization of articles was discussed with a third author (GvdH) until agreement was reached; the selection is therefore based on a full consensus.

## Results

### Journals

The 10 selected Otolaryngology journals can be found in Table 3 (impact factor 1.8 to 2.8). The scope of these journals can be found online (Appendix 2), two did not publish clinical evidence. The 20 selected medical journals had an impact factor varying from 6.0 to 101.8 (Table 3). A complete list of all evaluated medical journals is available online (Appendix 1). The selection process is shown in Figure 1. The titles of the selected journals can be found in Table 3.1 (Otolaryngology journals) and Table 3.2 (medical journals).

**Table 3.1:** Results 1. Type of research in Otolaryngology journals.

**Journal title**	**IF**	**Citable articles (% of total)**	**Clinical research (% of citable articles)**	**Systematic reviews (% of citable articles)**	**Therapy**	**Diagnosis**	**Prognosis**	**Etiology**	**Other**
Journal of the Association for Research in Otolaryngology	2.8	49 (3)	0 (0)	0 (0)	0	0	0	0	0
Hearing Research	2.7	178 (13)	20 (11)	0	2	0	1	4	13
Ear and hearing	2.6	80 (5)	50 (63)	0	4	1	2	4	39
Audiology & Neurotology	2.5	44 (3)	19 (43)	0	7	1	1	3	7
Head & Neck	2.4	209 (14)	129 (62)	6 (3)	61	19	34	4	11
Clinical Otolaryngology	2.4	54 (4)	38 (70)	7 (13)	15	3	4	1	15
Rhinology	2.3	118 (8)	82 (69)	1 (1)	28	5	10	22	17
Laryngoscope	2.0	422 (28)	214 (51)	7 (2)	89	10	22	42	51
Otology & Neurotology	1.9	260 (17)	142 (55)	6 (2)	56	2	14	15	55
Current Opinion in Otolaryngology	1.8	86 (6)	0	0	0	0	0	0	0
Total	–	1500	694 (46)	27 (2)	262 (38)	41 (6)	88 (13)	95 (14)	208 (30)

*IF, impact factor; %, percentage*.

**Table 3.2:** Results 2. Type of research in selected medical journals.

**Journal title**	**IF**	**Citable articles**	**Clinical research^**$**^ (% of citable articles)**	**Systematic reviews (% of citable articles)**	**Therapy**	**Diagnosis**	**Prognosis**	**Etiology**	**Other**
CA a cancer journal for clinicians	101.8	18	0	0	–	–	–	–	–
New England journal of medicine	53.3	345	1 (0.3)	0	0	0	1	0	0
Lancet	38.3	271	0	0	–	–	–	–	–
JAMA	30.1	233	3 (1.3)	1 (0.4)	2	0	0	0	1
Lancet Oncology	22.6	108	3 (2.8)	0	3	0	0	0	0
Journal of Clinical Oncology	18.4	784	21 (2.7)	0	11	4	5	0	1
Annals of Internal Medicine	16.7	167	0	0	0	0	0	0	0
Plos Medicine	16.3	99	0	0	–	–	–	–	–
British Medical Journal	14.1	312	1 (0.3)	0	1	0	0	0	0
Journal of the National Cancer Institute	13.8	135	1 (0.7)	0	1	0	0	0	0
Archives of Internal Medicine/ JAMA Internal Medicine	11.5	204	0	0	0	0	0	0	0
American Journal of Respiratory and Critical Care Medicine	11.1	310	6 (1.9)	0	1	0	0	4	1
Journal of Allergy and Clinical Immunology	11.0	336	14 (4.2)	1 (0.3)	6	0	0	7	1
Canadian Medical Association Journal	8.2	123	0	0	–	–	–	–	–
Clinical Cancer research	7.7	629	18 (2.9)	0	5	0	9	3	1
Annals of Surgery	7.5	291	2 (0.7)	0	0	0	1	0	1
American Journal of Clinical Nutrition	6.7	389	2 (0.5)	0	2	0	0	0	0
Annals of Oncology	6.4	445	12 (2.7)	0	6	1	3	0	2
Allergy	6.1	185	18 (9.7)	0	2	0	2	8	6
BMC Med	6.0	78	0	0	0	0	0	0	0
Total (%)		10,967	102 (0.9)	2 (0.02)	40 (39)	5 (5)	21 (21)	22 (22)	14 (14)

*IF, impact factor; $, related to Otolaryngology; %, percentage*.

### Publications

**Figure 2:**
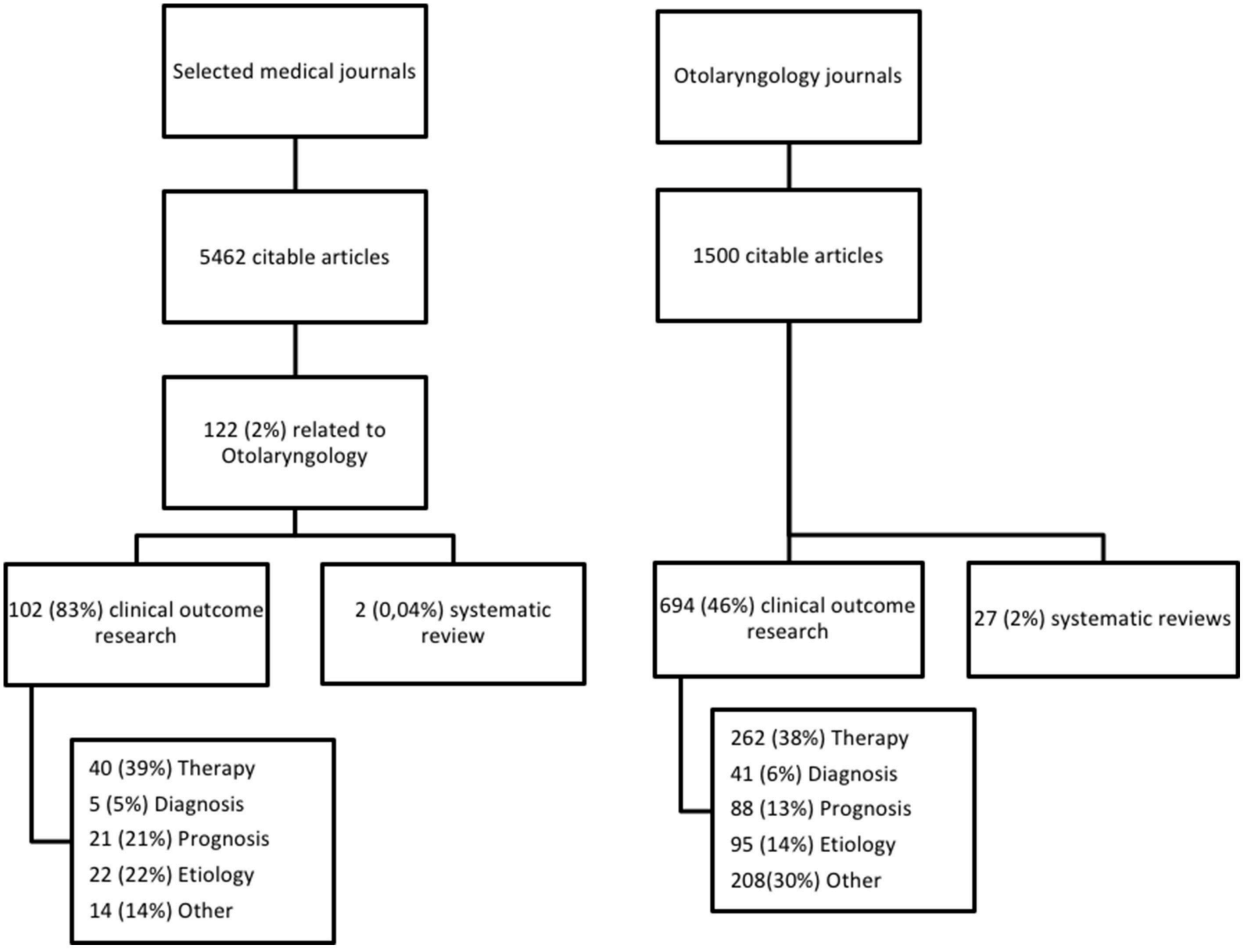
Selection of publications.

From 1,500 articles in Otolaryngology journals, we identified 694 (46%) original publications on clinical research and 27 (2%) systematic reviews (Figure 2). Of 5,462 citable articles in selected medical journals, 122 (2%) were related to Otolaryngology. Of these, 102 (83%) were original publications and 2 (0,04%) were systematic reviews. The different research questions are shown in Figure 2. Most publications concern therapy, followed by prognosis and etiology, least represented is diagnosis. The proportion of publications on prognosis and etiology research is lower in Otolaryngology journals. For diagnosis and therapy there are no differences. The results per journal can be found in Tables 3.1,3.2. The variation in the proportion of clinical research between journals could be explained by the different scopes of the journals; JARO only publishes basic research and Current Opinion in Otolaryngology only publishes (non-systematic) reviews (see online Appendix 2). If we exclude these journals from the analysis, the proportion of clinical research increases to 51%.

## Discussion

### Synopsis of Key Findings

We set out to find the amount of clinical research in Otolaryngology, published both in leading Otolaryngology journals and in medical journals outside this field.

Two percent of Otolaryngology related research is published in journals outside the field of Otolaryngology. In selected Otolaryngology journals, 46% (95% confidence interval 44; 49) of publications relates to clinical research. The proportion increased to 51% (95% confidence interval 48; 53) after excluding two journals that do not publish clinical research.

We found that the impact factor of medical journals outside the field of Otolaryngology (6.0 to 101.8) is higher than the impact factor of Otolaryngology journals (1.8 to 2.8).

Regarding research questions, 40% of publications was related to therapy.

### Comparison to Previous Studies

The proportion of clinical research in Otolaryngology is lower than the 75% reported previously (5–7) The difference may be explained by selection of different journals (the only identical journal included in the previous studies was the Laryngoscope), by our use of possible more stringent definitions than in previous research. It could also indicate an actual decrease in clinical evidence. Compared to other specialties, Otolaryngology journals achieve similar or higher rates of clinical evidence. For example, previous studies found an amount of clinical evidence in Urology journals of 35% (2002–2010) (9), 53% in anesthetics (2000–2009), (10) 11% in plastic surgery (2002) (11) and 24% in ophthalmology (2002) (12).

### Limitations of Our Study

We used the impact factor of 2011, which relates to publications from 2010 and 2011, but for this study we only selected publications from 2010. We selected 30 medical journals and reviewed over 300 journals. These journals were selected retrospectively, therefore selection bias could have occurred. The selected publications can be judged outdated. However, it takes some time for studies to become available full text and to be indexed. Then it also takes several years before studies are adopted in daily practice (13). When we look at clinical practice guidelines, it is common to find references of studies of 2010 and before. Therefore, our results are still important and informative. We selected articles based on the impact factor of the journal they were published in, since we wanted to show results for leading journals, since they are often read and looked to for relevant research. With this selection approach, we systematically evaluated almost 12,500 articles. However, we might have missed publications with our search strategy, so the actual amount of clinical outcome research could be either lower or higher.

### Implications for Clinicians and Researchers

For Otolaryngology journals, it is striking that some high impact factor journals do not strive to publish clinical research. In addition, our results show that 2% of Otolaryngology related clinical outcome research is published in journals outside the field of Otolaryngology. These findings supports results from different specialties, i.e., that important clinical studies are often not published in specialty journals (14). Moreover, Otolaryngology related research of substantial quality might be published in medical journals outside the field of Otolaryngology, since higher impact factors can be achieved. Doctors should therefore look beyond their specialized journals when searching for evidence. Forty percent of publications we found, report about therapy, which is a similar result to previous studies (7). Studies concerning etiology, prognosis and diagnosis are less common. This implies that the emphasis of researchers and journals is more on therapy than diagnosis, prognosis or etiology. Yet they should realize that these purpose categories are also important for clinical practice (3). Our results show a limited number of systematic reviews (both in and outside of Otolaryngology journals). Systematic reviews are of high importance since they sum op the results of existing studies (4). Therefore, we highly recommend that the amount of systematic reviews should increase, both in and outside of Otolaryngology journals.

For evidence-based practice, clinical original studies are also of vital importance (1, 2). We must therefore also ensure an increase of this type of research, to improve and optimize medical practice (15). This applies particularly to Otolaryngology journals, journals outside the field of Otolaryngology showed a better balance between clinical and non-clinical (e.g. fundamental) research (50 vs. 83%).

The amount of clinical evidence is predominantly determined by the scope of a journal and the choice of the editors and reviewers, but also by amount of studies that are conducted and submitted (15). On one hand, this implies that editors and reviewers of journals should watch for balance between publication of clinical and fundamental research. On the other hand, researchers and doctors involved in research, are encouraged to publish clinical evidence (15).

## Author Contributions

NK conception and design, acquisition of data, analysis and interpretation of data, drafting and revising the article, final approval of the version to be submitted and any revised version. GR acquisition of data, analysis and interpretation of data, drafting the article, final approval of the version to be submitted and any revised version. MA analysis and interpretation of data, revising the article, final approval of the version to be submitted and any revised version. GvdH conception and design, analysis and interpretation of data, drafting and revising the article, final approval of the version to be submitted and any revised version.

### Conflict of Interest Statement

The authors declare that the research was conducted in the absence of any commercial or financial relationships that could be construed as a potential conflict of interest.
