# Supplementary material for: Publications on Clinical Research in Otolaryngology–A Systematic Analysis of Leading Journals in 2010
Source: Front Surg. 2019 Apr 9;6:18. doi: 10.3389/fsurg.2019.00018 (PMC6467099; doi:10.3389/fsurg.2019.00018)
Supplement: Supplementary file 1 [file Table_1.DOCX]

**Appendix 1. List of all journals listed in the InCites Journal Citation Reports 2011, with impact factor ranging from high to low ^11^**

|  | Journal Title | IF | Medical journal | Subspecialty related to otolaryngology |
| --- | --- | --- | --- | --- |
|  | **CA-A CANCER JOURNAL FOR CLINICIANS** | **101.8** | **yes** | **yes** |
|  | **NEW ENGLAND JOURNAL OF MEDICINE** | **53.3** | **yes** | **yes** |
|  | ANNUAL REVIEW OF IMMUNOLOGY | 52.8 | yes | no |
|  | REVIEWS OF MODERN PHYSICS | 42.9 | no | - |
|  | CHEMICAL REVIEWS | 40.2 | no | - |
|  | NATURE REVIEWS MOLECULAR CELL BIOLOGY | 39.1 | no | - |
|  | **LANCET** | **38.3** | **yes** | **yes** |
|  | NATURE REVIEWS GENETICS | 38.1 | no | - |
|  | NATURE REVIEWS CANCER | 37.5 | no | - |
|  | ADVANCES IN PHYSICS | 37.0 | no | - |
|  | NATURE | 36.3 | no | - |
|  | NATURE GENETICS | 35.5 | no | - |
|  | ANNUAL REVIEW OF BIOCHEMISTRY | 34.3 | no | - |
|  | NATURE REVIEWS IMMUNOLOGY | 33.2 | no | - |
|  | NATURE MATERIALS | 32.8 | no | - |
|  | CELL | 32.4 | no | - |
|  | ENERGY EDUCATION SCIENCE AND TECHNOLOGY | 31.7 | no | - |
|  | SCIENCE | 31.2 | no | - |
|  | NATURE REVIEWS NEUROSCIENCE | 30.4 | no | - |
|  | **JAMA-JOURNAL OF THE AMERICAN MEDICAL ASSOCIATION** | **30.0** | **yes** | **yes** |
|  | NATURE PHOTONICS | 29.3 | no | - |
|  | NATURE REVIEWS DRUG DISCOVERY | 29.0 | no | - |
|  | CHEMICAL SOCIETY REVIEWS | 28.8 | no | - |
|  | NATURE NANOTECHNOLOGY | 27.3 | no | - |
|  | PHYSIOLOGICAL REVIEWS | 26.9 | no | - |
|  | CANCER CELL | 26.6 | no | - |
|  | ANNUAL REVIEW OF ASTRONOMY AND ASTROPHYSICS | 26.5 | no | - |
|  | NATURE IMMUNOLOGY | 26.0 | yes | no |
|  | ANNUAL REVIEW OF PLANT BIOLOGY | 25.96 | no | - |
|  | ANNUAL REVIEW OF NEUROSCIENCE | 25.7 | no | - |
|  | CELL STEM CELL | 25.4 | no | - |
|  | BEHAVIORAL AND BRAIN SCIENCES | 25.1 | no | - |
|  | PROGRESS IN POLYMER SCIENCES | 24.1 | no | - |
|  | LANCET NEUROLOGY | 23.5 | yes | no |
|  | LANCET BIOTECHNOLOGY | 23.3 | no | - |
|  | NATURE BIOTECHNOLOGY | 23.3 | no | - |
|  | **LANCET ONCOLOGY** | **22.6** | **yes** | **yes** |
|  | NATURE MEDICINE | 22.5 | yes | no |
|  | ANNUAL REVIEWS OF GENETICS | 22.2 | yes | no |
|  | ACCOUNTS OF CHEMICAL RESEARCH | 21.6 | no | - |
|  | ANNUAL REVIEW OF PHARMACOLOGY AND TOXICOLOGY | 21.6 | no | - |
|  | IMMUNITY | 21.6 | yes | no |
|  | NATURE REVIEWS MICROBIOLOGY | 21.2 | no | - |
|  | ANNUAL REVIEW OF PHYSIOLOGY | 20.8 | no | - |
|  | NATURE CHEMISTRY | 20.5 | no | - |
|  | PHYSICS REPORTS | 20.4 | no | - |
|  | ANNUAL REVIEW OF PATHOLOGY | 20.0 | yes | no |
|  | ENDOCRINE REVIEWS | 19.9 | yes | no |
|  | NATURE CELL BIOLOGY | 19.5 | no | - |
|  | NATURE METHODS | 19.3 | no | - |
|  | NATURE PHYSICS | 19.0 | no | - |
|  | **JOURNAL OF CLINICAL ONCOLOGY** | **18.4** | **yes** | **yes** |
|  | PROGRESS IN MATERIALS SCIENCE | 18.2 | no | - |
|  | ECOLOGY LETTERS | 17.6 | no | - |
|  | LIVING REVIEWS IN RELATIVITY | 17.5 | no | - |
|  | LANCET INFECTIOUS DISEASES | 17.5 | no | - |
|  | ANNUAL REVIEW OF PSYCHOLOGY | 16.8 | yes | no |
|  | **ANNALS OF INTERNAL MEDICINE** | **16.7** | **yes** | **yes** |
|  | ANNUAL REVIEW OF MARINE SCIENCE | 16.5 | no | - |
|  | **PLOS MEDICNE** | **16.3** | **yes** | **yes** |
|  | CLINICAL MICROBIOLOGY REVIEWS | 16.1 | yes | no |
|  | ALDRICHIMICA ACTA | 16.1 | no | - |
|  | ANNUAL REVIEW OF CELL EN DEVELOPMENTAL BIOLOGY | 15.8 | no | - |
|  | TRENDS IN ECOLOGY&EVOLUTION | 15.8 | no | - |
|  | NATURE NEUROSCIENCE | 15.5 | no | - |
|  | NANO TODAY | 15.4 | no | - |
|  | MATERIALS SCIENCE & ENGINEERING R-REPORTS | 15.0 | no | - |
|  | ANNUAL REVIEW OF GENOMICS AND HUMA GENETICS | 14.8 | no | - |
|  | CIRCULATION | 14.7 | yes | no |
|  | NEURON | 14.7 | no | - |
|  | REPORTS ON PROGRESS IN PHYSICS | 14.7 | no | - |
|  | NATURE CHEMICAL BIOLOGY | 14.7 | no | - |
|  | PSYCHOLOGICAL BULLETIN | 14.5 | no | - |
|  | ANNUAL REVIEW OF ECOLOGY EVOLUTION AND SYSTEMATICS | 14.4 | no | - |
|  | ANNUAL REVIEW OF MICROBIOLOGY | 14.3 | yes | no |
|  | TRENDS IN NEUROSCIENCES | 14.2 | no |  |
|  | PROGRESS IN ENERGY AND COMBUSTION SCIENCE | 14.2 | no | - |
|  | MOLECULAR CELL | 14.2 | no | - |
|  | JOURNAL OF THE AMERICAN COLLEGE OF CARDIOLOGY | 14.2 | yes | no |
|  | ANNUAL REVIEW OF PHYSICAL CHEMISTRY | 14.1 | no | - |
|  | **BRITISCH MEDICAL JOURNAL** | **14.1** | **yes** | **yes** |
|  | DEVELOPMENTAL CELL | 14.1 | no | - |
|  | ADVANCED MATERIALS | 14.0 | no | - |
|  | JOURNAL OF EXPIRIMENTAL MEDICINE | 13.9 | yes | no |
|  | **JOURNAL OF THE NATIONAL CANCER INSTITUTE** | **13.8** | **yes** | **yes** |
|  | CELL METABOLISM | 13.7 | no | - |
|  | MOLECULAR PSYCHIATRY | 13.7 | no | - |
|  | GENOME RESEARCH | 13.6 | no | - |
|  | ANNUAL REVIEW OF BIOPHYSICS | 13.6 | no | - |
|  | CELL HOST & MICROBE | 13.5 | no | - |
|  | ASTROPHYSICAL JOURNAL | 13.5 | no | - |
|  | ANGEWANDTE CHEMIE- INTERNATIONAL EDITION | 13.5 | no | - |
|  | NANO LETTERS | 13.2 | no | - |
|  | ANNUAL REVIEW OF MEDICINE | 13.1 | yes | no |
|  | ANNUAL REVIEW OF MATERIALS RESEARCH | 13.1 | no | - |
|  | JOURNAL OF CLINICAL INVESTIGATION | 13.1 | yes | no |
|  | MICROBIOLOGY AND MOLECULA BIOLOGY REVIEWS | 13.0 | no | - |
|  | CURRENT OPINION IN CELL BIOLOGY | 12.9 | no | - |
|  | ANNUAL REVIEW OF FLUID MECHANICS | 12.8 | no | - |
|  | NATURE STRUCTURAL & MOLECULAR BIOLOGY | 12.7 | no | - |
|  | ACTA CRISTALLOGRAPHICA | 12.6 | no | - |
|  | TRENDS IN COGNITIVE SCIENCES | 12.6 | no | - |
|  | AMERICAN JOURNAL OF PSYCHIATRY | 12.5 | yes | no |
|  | LIVING REVIEWS IN SOLAR PHYSICS | 12.5 | no | - |
|  | NATURE REVIEWS NEUROLOGY | 12.5 | yes | no |
|  | ANNUAL REVIEW OF CONDENSED MATTER PHYSICS | 12.4 | no | - |
|  | REVIEWS OF GEOPHYSICS | 12.4 | no | - |
|  | TRENDS IN CELL BIOLOGY | 12.4 | no | - |
|  | ANNUAL REVIEW OF BIOMEDICAL ENGINEERING | 12.2 | no | - |
|  | COORDINATION CHEMISTRY REVIEWS | 12.1 | no | - |
|  | ARCHIVES OF GENERAL PSYCHIATRY | 12.0 | yes | no |
|  | NATURE REVIEWS CLINICAL ONCOLOGY | 12.0 | yes | no |
|  | NATURE GEOSCIENCE | 11.8 | no | - |
|  | SURFACE SCIENCE REPORTS | 11.7 | no | - |
|  | GASTROENTEROLOGY | 11.7 | yes | no |
|  | HEPATOLOGY | 11.7 | yes | no |
|  | GENES AND DEVELOPMENT | 11.7 | no | - |
|  | ASTRONOMY AND ASTRPHYSICS REVIEW | 11.5 | no | - |
|  | ADVANCED DRUG DELIVERY REVIEWS | 11.5 | no | - |
|  | **ARCHIVES OF INTERNAL MEDICINE** | **11.5** | **yes** | **yes** |
|  | ANNUAL REVIEW OF ENTOMOLOGY | 11.5 | no | - |
|  | PLOS BIOLOGY | 11.5 | no | - |
|  | FRONTIERS IN NEUROENDOCRINOLOGY | 11.4 | yes | no |
|  | ACS NANO | 11.4 | no | - |
|  | IMMUNOLOGICAL REVIEWS | 11.1 | no | - |
|  | ANNALS OF NEUROLOGY | 11.1 | yes | no |
|  | **AMERICAN JOURNAL OF RESPIRATORY AND CRITICAL CARE MEDICINE** | **11.1** | **yes** | **yes** |
|  | TRENDS IN PLANT SCIENCE | 11.0 | no | no |
|  | **JOURNAL OF ALLERGY AND CLINICAL IMMUNOLOGY** | **11.0** | **yes** | **yes** |
|  | FEMS MICROBIOLOGY REVIEWS | 11.0 | no | - |
|  | TRENDS IN PHARMACOLOGICAL RESEARCH | 10.9 | no | - |
|  | TRENDS IN BIOCHEMICAL SCIENCES | 10.8 | no | - |
|  | PAIN PHYSICIAN | 10.7 | yes | no |
|  | MEDICINAL RESEARCH REVIEWS | 10.7 | yes | no |
|  | PROGRESS IN LIPID RESEARCH | 10.7 | no | - |
|  | STUDIES IN MYCOLOGY | 10.6 | no | - |
|  | AMERICAN JOURNAL OF HUMAN GENETICS | 10.6 | no | - |
|  | CANCER AND METASTASIS REVIEWS | 10.6 | yes | no |
|  | EUROPEAN HEART JOURNAL | 10.5 | yes | no |
|  | MASS SPECTOMETRY REVIEWS | 10.5 | no | - |
|  | TRENDS IN IMMUNOLOGY | 10.4 | yes | no |
|  | JOURNAL OF PHOTOCHEMISTRY AND PHOTOBIOLOGY C-PHOTOCHEMISTRY REVIEWS | 10.4 | no | - |
|  | TRENDS IN MOLECULAR MEDICINE | 10.4 | no | - |
|  | BRAIN RESEARCH REVIEWS | 10.3 | no | - |
|  | EMBO MOLECULA MEDICINE | 10.3 | no | - |
|  | JOURNAL OF CELL BIOLOGY | 10.3 | no | - |
|  | SYSTEMATIC BIOLOGY | 10.2 | no | - |
|  | ADVANCED FUNCTIONAL MATERIALS | 10.2 | no | - |
|  | GUT | 10.1 | yes | no |
|  | QUARTERLY REVIEWS OF BIOPHYSICS | 10.1 | no | - |
|  | TRENDS IN GENETICS | 10.1 | no | - |
|  | SIAM REVIEWS | 10.0 | no | - |
|  | NATURE REVIEWS ENDOCRINOLOGY | 10.0 | yes | no |
|  | LASER PHYSICS LETTERS | 10.0 | no | - |
|  | MOLECULAR ASPECTS OF MEDICINE | 10.0 | no | - |
|  | NATURE PROTOCOLS | 10.0 | no | - |
|  | JOURNAL OF THE AMERICAN CHEMICAL SOCIETY | 10.0 | no | - |
|  | BLOOD | 9.9 | yes | no |
|  | ADVANCES IN MICROBIAL PHYSIOLOGY | 9.9 | no | - |
|  | ANNUAL REVIEW OF PHYTOPATHOLOGY | 9.9 | no | - |
|  | CURRENT OPINION IN CHEMICAL BIOLOGY | 9.9 | no | - |
|  | NATURAL PRODUCT REPORTS | 9.8 | no | - |
|  | PROCEEDING OF THE NATIONAL ACADEMY OF THE UNITED STATES OF AMERICA | 9.7 | no | - |
|  | JOURNAL OF THE AMERICAN SOCIET OF NEPHROLOGY | 9.7 | yes | no |
|  | CURRENT BIOLOGY | 9.6 | no | - |
|  | BIOTECHNOLOGY ADVANCES | 9.6 | no | - |
|  | ENERGY AND ENVIRONMENTAL SCIENCE | 9.6 | no | - |
|  | LEUKEMIA | 9.6 | yes | no |
|  | DRUG RESISTANCE UPDATES | 9.6 | no | - |
|  | CURRENT OPINION IN IMMUNOLOGY | 9.5 | yes | no |
|  | CIRCULATION RESEARCH | 9.5 | yes | no |
|  | CRITICAL REVIEWS IN SOLID STATE AND MATERIAL SCIENCES | 9.5 | no | - |
|  | BRAIN | 9.5 | yes | no |
|  | PROGRESS IN RETINAL AND EYE RESEARCH | 9.5 | yes | no |
|  | ANNUAL REVIEW OF NUTRITION | 9.4 | yes | no |
|  | CURRENT OPINION IN STRUCTURAL BIOLOGY | 9.4 | no | - |
|  | COLD SPRING HARBOUR PERSPECTIVES IN BIOLOGY | 9.4 | no | - |
|  | ACTA-REVIEWS ON CANCER | 9.4 | yes | no |
|  | PROGRESS IN INORGANIC CHEMISTRY | 9.3 | no | - |
|  | ACTA BEUROPATHOLOGICA | 9.3 | no | - |
|  | CURRENT OPINION IN PLANT BIOLOGY | 9.3 | no | - |
|  | JOURNAL OF HEPATOLOGY | 9.3 | yes | no |
|  | JOURNAL OF ECONOMIC LITERATURE | 9.2 | no | - |
|  | HUMAN REPORDUCTION UPDATE | 9.2 | no | - |
|  | EMBO JOURNAL | 9.2 | no | - |
|  | CLINICAL INFECTIOUS DISEASES | 9.2 | yes | no |
|  | TRENDS IN BIOTECHNOLOGY | 9.1 | no | - |
|  | PLOS PATHOGENS | 9.1 | no | - |
|  | FRONTIERS IN ECOLOGY AND THE ENVIRONMENT | 9.1 | no | - |
|  | ANNUAL REVIEW OF CLINICAL PSYCHOLOGY | 9.1 | yes | no |
|  | BIOLOGICAL REVIEWS | 9.1 | no | - |
|  | ANNUAL REVIEW OF ANALYTICAL CHEMISTRY | 9.0 | no | - |
|  | GENOME BIOLOGY | 9.0 | no | - |
|  | PLANT CELL | 9.0 | no | - |
|  | PROGRESS IN NEUROBIOLOGY | 8.9 | no | - |
|  | CELL DEATH AND DIFFERENTIATION | 8.8 | no | - |
|  | NATURE REVIEWS CARDIOLOGY | 8.8 | yes | no |
|  | SCHIZOFRENIA BULLETIN | 8.8 | yes | - |
|  | ANNALS OF THE REUMATIC DISEASE | 8.7 | yes | no |
|  | PLOS GENETICS | 8.7 | no | - |
|  | NEUROSCIENCE AND BIOBEHAVIOURAL REVIEWS | 8.7 | no | - |
|  | PROGRESS IN SURFACE SCIENCE | 8.6 | no | - |
|  | MOLECULAR SYSTEMS BIOLOGY | 8.6 | no | - |
|  | PHARMACOLOGY AND THERAPEUTICS | 8.6 | no | - |
|  | EUROPEAN UROLGY | 8.5 | yes | no |
|  | ANTIOXIDANT & REDOX SIGNALING | 8.5 | no | - |
|  | NATURE REVIEWS RHEUMATOLOGY | 8.4 | yes | no |
|  | SMALL | 8.3 | no | - |
|  | NEUROLOGY | 8.3 | yes | no |
|  | DIABETES | 8.3 | yes | no |
|  | BIOLOGICAL PSYCHIATRY | 8.3 | yes | no |
|  | **CANADIAN MEDICAL ASSOCIATION JOURNAL** | **8.2** | **yes** | **yes** |
|  | ADVANCES IN COLLOID AND INTERFACE SCIENCE | 8.1 | no | - |
|  | TREND IN ENDOCRINOLGY AND METABOLISM | 8.1 | yes | no |
|  | NATURE REVIEWS GASTROENTEROLOGY AND HEPATOLOGY | 8.1 | yes | no |
|  | DIABETES CARE | 8.1 | yes | no |
|  | CURRENT OPINION IN GENETICS AND DEVELOPMENT | 8.1 | no | - |
|  | NUCLEIC ACIDS RESEARCH | 8.0 | no | - |
|  | CURRENT OPINION IN COLLOID AND INTERFACE SCIENCE | 8.0 | no | - |
|  | NATURE CLINICAL PRACTICE ONCOLOGY | 8.0 | yes | no |
|  | NEURPSYCHOPHARMACOLOGY | 8.0 | no | - |
|  | PHYSIOLOGY | 8.0 | no | - |
|  | CURRENT OPINION IN MICROBIOLOGY | 7.9 | no | - |
|  | TRENDS IN MICROBIOLOGY | 7.9 | no | - |
|  | CLINICAL CHEMISTRY | 7.9 | no | - |
|  | ARTHRITIS AND RHEUMATISM | 7.9 | yes | no |
|  | CANCER RESEARCH | 7.9 | yes | no |
|  | CYTOKINE & GROWTH FACTOR REVIEWS | 7.8 | no | - |
|  | SCIENCE TRANSLATIONAL MEDICINE | 7.8 | no | - |
|  | STEM CELLS | 7.8 | no | - |
|  | PSYCHOLOGICAL REVIEW | 7.8 | no | - |
|  | **CLINICAL CANCER RESEARCH** | **7.7** | **yes** | **yes** |
|  | QUARTERLY REVIEW OF BIOLOGY | 7.7 | no | - |
|  | CURRENT OPINION IN BIOTECHNOGLY | 7.7 | no | - |
|  | JOURNAL OF MOLECULAR CELL BIOLOGY | 7.6 | no | - |
|  | CRITICAL REVIEWS IN BIOCHEMISTRY | 7.6 | no | - |
|  | HUMAN MOLECULAR GENETICS | 7.6 | no | - |
|  | NATURE CLINICAL PRACTICE NEUROLOGY | 7.6 | yes | no |
|  | ARCHIVES OF NEUROLOGY | 7.6 | yes | no |
|  | EPIDEMIOLOGIC REVIEWS | 7.6 | yes | no |
|  | NATURE CLINICAL PRACTICE ENDOCRINOLOGY&METABOLISM | 7.5 | yes | no |
|  | CHEMICAL SCIENCE | 7.5 | no | - |
|  | CATALYSIS REVIEWS | 7.5 | no | - |
|  | SCIENCE SIGNALING | 7.5 | no | - |
|  | **ANNALS OF SURGERY** | **7.5** | **yes** | **yes** |
|  | AUTOPHAGY | 7.5 | yes | no |
|  | CURRENT OPINION IN NEUROBIOLOGY | 7.4 | no | - |
|  | ECOLOGICAL MONOGRAPHS | 7.4 | no | - |
|  | BIOMATERIALS | 7.4 | no | - |
|  | MOLECULAR AND CELLULAR PROTEOMICS | 7.4 | no | - |
|  | NATURE COMMUNICATION | 7.4 | no | - |
|  | LASER AND PHOTONICS REVIEWS | 7.4 | no | - |
|  | ISME JOURNAL | 7.4 | no | - |
|  | PHYSICAL REVIEW LETTERS | 7.4 | no | - |
|  | JOURNAL OF AUTOIMMUNITY | 7.4 | yes | no |
|  | EMBO REPORTS | 7.4 | no | - |
|  | BASIC RESEARCH IN CARDIOLOGY | 7.3 | no | - |
|  | ANNUAL REVIEW OF CHEMICAL AND BIOMOLECULAR ENGINEERING | 7.3 | no | - |
|  | CHEMISTRY OF MATERIALS | 7.3 | no | - |
|  | AMERICAN JOURNAL OF GASTROENTEROLOGY | 7.3 | yes | no |
|  | PARTICLE AND FIBER TOCICOLOGY | 7.3 | no | - |
|  | ANNUAL REVIEW OF EARTH AND PLANETARY SCIENCES | 7.2 | no | - |
|  | PHYSICS OF LIFE REVIEWS | 7.2 | no | - |
|  | REVIEWS IN MEDICAL VIROLOGY | 7.2 | no | - |
|  | EXPERT REVIEWS IN MOLECULAR MEDICINE | 7.1 | no | - |
|  | JOURNAL OF NEUROSCIENCE | 7.1 | yes | no |
|  | NATURE REVIEWS NEPHROLOGY | 7.1 | yes | no |
|  | INTERNATIONAL JOURNAL OF CARDIOLOGY | 7.1 | yes | no |
|  | CLINICAL PSYCHOLOGY REVIEW | 7.1 | yes | no |
|  | SEMINARS IN LIVER DISEASE | 7.1 | yes | no |
|  | NATURE CLINICAL PRACTICE CARDIOVASCULAR MEDICINE | 7.0 | yes | no |
|  | OBESITY REVIEWS | 7.0 | yes | no |
|  | ENVIRONMENTAL HEALTH PERSEPCTIVES | 7.0 | no | - |
|  | ADVANCES IN ORGANOMETALLIC CHEMISTRY | 7.0 | no | - |
|  | PROGRESS IN QUENSTUM ELECTRONIS | 7.0 | no | - |
|  | NANO RESEARCH | 7.0 | no | - |
|  | MUCOSAL IMMUNOLOGY | 7.0 | no | - |
|  | INTERNATIONAL MATERIALS REVIEWS | 7.0 | no | - |
|  | SLEEP MEDICINE REVIEWS | 6.9 | yes | no |
|  | MOLECULAR THERAPY | 6.9 | no | - |
|  | AMERICAN PSYCHOLOGIST | 6.9 | yes | no |
|  | GLOBAL ENVIRONMENTAL CHANGE | 6.9 | no | - |
|  | CURRENT OPINION IN PHARMACOLOGY | 6.9 | no | - |
|  | THORAX | 6.8 | yes | no |
|  | DRUG DISCOVERY TODAY | 6.8 | no | - |
|  | CHEMS SUS CHEM | 6.8 | no | - |
|  | DIABETOLOGIA | 6.8 | yes | no |
|  | PROCEDINGS OF THE IEEE | 6.8 | no |  |
|  | JACC-CARDIOVASCULAR INTERVENTIONS | 6.8 | yes | no |
|  | JOURNAL OF MAMMARY GLAND BIOLOGY AND NEOPLASIA | 6.7 | no | - |
|  | ADVANCES IN CANCER RESEARCH | 6.7 | yes | no |
|  | NANOMEDICINE | 6.7 | no | - |
|  | **AMERICAN JOURNAL OF CLINICAL NUTRITION** | **6.7** | **yes** | **yes** |
|  | GONDWANA RESEARCH | 6.7 | no | - |
|  | SEMINARS IN CELL&DEVELOPMENTAL BIOLOGY | 6.6 | no | - |
|  | NEW PHYTOLOGIST | 6.6 | no | - |
|  | AUTOIMMUNITY REVIEWS | 6.6 | no | - |
|  | BRITISH JOURNAL OF PSYCHIATRY | 6.6 | yes | no |
|  | NEUROPSYCHOLOGY REVIEW | 6.6 | yes | no |
|  | KIDNEY INTERNATIONAL | 6.6 | yes | no |
|  | DEVELOPMENT | 6.6 | no | - |
|  | EARTH-SCIENCE REVIEWs | 6.6 | no | - |
|  | CELLULAR AND MOLECULAR LIFE SCIENCES | 6.6 | no | - |
|  | TOPICS IN CURRENT CHEMISTRY | 6.6 | no | - |
|  | CEREBRAL CORTES | 6.5 | no | - |
|  | PLANT PHYSIOLOGY | 6.5 | no | - |
|  | JOURNAL OF CONTROLLED RELEASE | 6.5 | no | - |
|  | SEMINARS IN CANCER BIOLOGY | 6.5 | no | - |
|  | CRITICAL REVIEWS IN BIOTECHNOLOGY | 6.5 | no | - |
|  | RETROVIROLOGY | 6.5 | no | - |
|  | CIRCULATION ARRHYTHMIA AND ELECTROPHYSIOLOGY | 6.5 | no | - |
|  | MUTATION RESEARCH | 6.5 | no | - |
|  | ANNUAL REVIEW OF NUCLEAR AND PARTICLE SCIENCE | 6.5 | no | - |
|  | ACS CHEMICAL BIOLOGY | 6.4 | no | - |
|  | JOURNAL OF THE AMERICAN OF CHILD AND ADOLESCENT PSYCHIATRY | 6.4 | yes | no |
|  | **ANNALS OF ONCOLOGY** |  | **yes** | **yes** |
|  | HAEMATOLOGICA | 6.4 | yes | no |
|  | ANNUAL REVIEW OF ENVIRONMENT AND RESOURCES | 6.4 | no | - |
|  | INTERNATIONAL JOURNAL OF EPIDEMIOLOGY | 6.4 | yes | no |
|  | JOURNAL OF INFECTIOUS DISEASES | 6.4 | yes | no |
|  | PHILOSOPHICAL TRANSACTIONS OF THE ROYAL SOCIETY B-BIOLOGICAL SCIENCES | 6.4 | no | - |
|  | DRUG METABOLISM REVIEW | 6.4 | no | - |
|  | AMERICAN JOURNAL OF TRANSPLANTATION | 6.4 | yes | no |
|  | SEMINARS IN IMMUNOLOGY | 6.4 | yes | no |
|  | JOURNAL OF NUCLEAR MEDICINE | 6.4 | no | - |
|  | ALZHEIMERS AND DEMENTIA | 6.4 | yes | no |
|  | JOURNAL OF BONE AND MINERAL RESEARCH | 6.4 | no | - |
|  | ONCOGENE | 6.4 | no | - |
|  | ARTERIOSCLEROSIS THROMBOSIS AND VASCULAR BIOLOGY | 6.4 | no | - |
|  | JOURNAL OF MEDICAL GENETICS | 6.4 | no | - |
|  | STRUCTURE | 6.3 | no | - |
|  | CRITICAL CARE MEDICINE | 6.3 | yes | no |
|  | GREEN CHEMISTRY | 6.3 | no | - |
|  | JOURNAL OF PATHOLOGY | 6.3 | no | - |
|  | JOURNAL OF INVESTIGATIVE DERMATOLOGY | 6.3 | yes | no |
|  | IEEE COMMUNICATION SURVEYS AND TUTORIALS | 6.3 |  | - |
|  | CIRCULATION HEART FAILURE | 6.3 | yes | no |
|  | PSYCHOTHERAPY AND PSCYHOSOMATICS | 6.3 | yes | no |
|  | POLYMER REVIEWS | 6.3 | no | - |
|  | SEMINARS IN IMMUNOPATHOLOGY | 6.3 | no | - |
|  | TRENDS IN ANALYTICAL CHEMISTRY | 6.3 | no | - |
|  | **ALLERGY** | **6.3** | **yes** | **yes** |
|  | CRITICAL REVIEWS IN MICROBIOLOGY | 6.3 | no | - |
|  | AGING CELL | 6.3 | no | - |
|  | AIDS | 6.2 | yes | no |
|  | WORLD PSYCHIATRY | 6.2 | yes | no |
|  | JOURNAL OF PHYSICAL CHEMISTRY LETTERS | 6.2 | no | - |
|  | HYPERTENSION | 6.2 | yes | no |
|  | NEUROBIOLOGY OF AGING | 6.2 | no | - |
|  | AGEING RESEARCH REVIEWS | 6.2 | no | - |
|  | ACADEMY OF MANAGEMENT REVIEW | 6.2 | no | - |
|  | CHEMICAL COMMUNICATIONS | 6.2 | no | - |
|  | EMERGING INFECTIOUS DISEASES | 6.2 | yes | no |
|  | PLANT JOURNAL | 6.2 | no | - |
|  | PSYCHOLOGICAL MEDICINE | 6.2 | yes | no |
|  | EUROSURVEILLANCE | 6.2 | yes | no |
|  | SOCIAL COGNITIVE AND AFFECTIVE NEUROSCIENCE | 6.1 | yes | no |
|  | JOURNAL OF CELL SCIENCE | 6.1 | no | - |
|  | CIRCULATION CARDIOVASCULAR GENETICS | 6.1 | no | - |
|  | BIOTECHNOLOGY FOR BIOFUELS | 6.1 | no | - |
|  | CURRENT OPINION IN LIPIDOLOGY | 6.1 | yes | no |
|  | NATURE CLINICAL PRACTICE NEPHROLOGY | 6.1 | yes | no |
|  | CORTEX | 6.1 | yes | no |
|  | PERSONALITY AND SOCIAL PSYCHOLOGY REVIEW | 6.1 | no | - |
|  | CARDIOVASCULAR RESERACH | 6.1 | yes | no |
|  | ANGIOGENESIS | 6.1 | yes | no |
|  | CIRCULATION CARDIOVACULAR INTERVENTIONS | 6.1 | yes | no |
|  | CANCER TREATMENT REVIEWS | 6.1 | yes | no |
|  | ADVANCED SYNTHESIS & CATALYSIS | 6.0 | no | - |
|  | CLINICAL PHARMACOLOGY AND THERAPEUTICS | 6.0 | no | no |
|  | **BMC MEDICINE** | **6.0** | **yes** | **yes** |

**Appendix 2. Scope for selected Otolaryngology journals**

| **Journal Title** | **Scope (as displayed on website of the journal)** |
| --- | --- |
| **1. Journal of the Association for Research in Otolaryngology (JARO)** | JARO is a peer-reviewed journal that publishes research findings focused on the auditory and vestibular systems. JARO welcomes submissions describing original experimental research that investigates the mechanisms underlying problems of basic or clinical significance. Clinical case studies, pharmaceutical screens and methods papers are not encouraged unless they include significant new findings as well. Commentaries and reviews are published at the discretion of the Editorial Board; consult the Editor-in-Chief before submitting |
| **2. Hearing Research** | The aim of the journal is to provide a forum for papers concerned with basic peripheral and central auditory mechanisms. Emphasis is on experimental and clinical studies, but theoretical and methodological papers will also be considered. The journal publishes original research papers, review and mini- review articles, rapid communications, method/protocol and perspective articles.  Papers submitted should deal with auditory anatomy, physiology, psychophysics, imaging, modeling and behavioural studies in animals and humans, as well as hearing aids and cochlear implants. Papers on comparative aspects of hearing and on effects of drugs and environmental contaminants on hearing function will also be considered. Clinical papers will be accepted when they contribute to the understanding of normal and pathological hearing functions. |
| **3. Ear and hearing** | From the basic science of hearing and balance disorders to auditory electrophysiology to amplification and the psychological factors of hearing loss, *Ear and Hearing* covers all aspects of auditory and vestibular disorders. This multidisciplinary journal consolidates the various factors that contribute to identification, remediation, and audiologic and vestibular rehabilitation. It is the one journal that serves the diverse interest of all members of this professional community -- otologists, audiologists, educators, and to those involved in the design, manufacture, and distribution of amplification systems. The original articles published in the journal focus on assessment, diagnosis, and management of auditory and vestibular disorders |
| **4. Audiology & Neurotology** | Audiology and Neurotology provides a forum for the publication of the most-advanced and rigorous scientific research related to the basic science and clinical aspects of the auditory and vestibular system and diseases of the ear. This journal seeks submission of cutting edge research opening up new and innovative fields of study that may improve our understanding and treatment of patients with disorders of the auditory and vestibular systems, their central connections and their perception in the central nervous system.  In addition to original papers the journal also offers invited review articles on current topics written by leading experts in the field.  The journal is of primary importance for all scientists and practitioners interested in audiology, otology and neurotology, auditory neurosciences and related disciplines |
| **5.Head & Neck** | Head & Neck is an international multidisciplinary publication of original contributions concerning the diagnosis and management of diseases of the head and neck. This area involves the overlapping interests and expertise of several surgical and medical specialties, including general surgery, neurosurgery, otolaryngology, plastic surgery, oral surgery, dermatology, ophthalmology, pathology, radiotherapy, medical oncology, and the corresponding basic sciences.  Head & Neck publishes original contributions on clinical and research topics. Each manuscript is submitted to peer review by at least two experts in the field. Comprehensive reviews of topics, particularly in fields subject to rapid change in knowledge, will be included at the discretion of the Editor and on the recommendation of reviewers. Technical notes, descriptions of new technologies, single case reports of unusual interest, and brief preliminary communications are accepted after proper peer review. |
| **6. Clinical Otolaryngology** | Clinical Otolaryngology is a bimonthly journal devoted to clinically-oriented research papers of the highest scientific standards dealing with:   - current otorhinolaryngological practice - audiology, otology, balance, rhinology, larynx, voice and paediatric ORL - head and neck oncology - head and neck plastic and reconstructive surgery - continuing medical education and ORL training   The emphasis is on high quality new work in the clinical field and on fresh, original research.  Each issue begins with an editorial expressing the personal opinions of an individual with a particular knowledge of a chosen subject. The main body of each issue is then devoted to original papers carrying important results for those working in the field. In addition, topical review articles are published discussing a particular subject in depth, including not only the opinions of the author but also any controversies surrounding the subject. |
| **7.Rhinology** | Review articles Reviews should present an update of the most recent developments in a particular field of rhinologic research research. We encourage the submission of high quality colour pictures and cartoons.  Original articles We welcome high quality original publications dealing with innovative aspects of rhinologic research.  Case Reports As from April 1, 2009, the journal does NOT accept anymore Case reports. |
| **8.Laryngoscope** | The Laryngoscope has been the leading source of information on advances in the diagnosis and treatment of head and neck disorders for nearly 120 years. The Laryngoscope is the first choice among otolaryngologists for publication of their important findings and techniques. Each monthly issue of The Laryngoscope features peer-reviewed medical, clinical, and research contributions in general otolaryngology, allergy/rhinology, otology/neurotology, laryngology/bronchoesophagology, head and neck surgery, sleep medicine, pediatric otolaryngology, facial plastics and reconstructive surgery, oncology, and communicative disorders. Contributions include papers and posters presented at the Annual and Section Meetings of the Triological Society, as well as independent papers, "How I Do It", "Triological Best Practice" articles, and contemporary reviews. Theses authored by the Triological Society’s new Fellows as well as papers presented at meetings of the American Laryngological Association are published in The Laryngoscope. |
| **9.Otology & Neurotology** | *​​​*Otology & Neurotology publishes original articles relating to both clinical and basic science aspects of otology, neurotology, and cranial base surgery. As the foremost journal in its field, it has become the favored place for publishing the best of new science relating to the human ear and its diseases. The broadly international character of its contributing authors, editorial board, and readership provides the Journal its decidedly global perspective. |
| **10.Current Opinion in Otolaryngology** | **​*​​***Current Opinion in Otolaryngology & Head and Neck Surgery is a bimonthly publication offering a unique and wide ranging perspective on the key developments in the field. Each issue features hand-picked review articles from our team of expert editors. With eleven disciplines published across the year – including maxillofacial surgery, head and neck oncology and speech therapy and rehabilitation – every issue also contains annotated references detailing the merits of the most important papers. |
